# IgA nephropathy with crescent cell lesions in a human brucellosis patient: a case report

**DOI:** 10.3389/fneph.2025.1594639

**Published:** 2025-08-26

**Authors:** Dongli Qi, Ricong Yu, Qijun Wan, Yi Xu

**Affiliations:** Department of Nephrology, Shenzhen Second People’s Hospital, The First Affiliated Hospital of Shenzhen University, Shenzhen, China

**Keywords:** glomerulonephritis, IgA nephropathy, brucellosis, acute kidney injury, renal biopsy pathology, *Brucella*-associated glomerular disease

## Abstract

Brucellosis is known to impact multiple organ systems in humans, including the urogenital system; however, the occurrence of glomerular diseases is relatively uncommon. In this study, we present the case of a 45-year-old man with no prior history of renal disease who developed gross hematuria, proteinuria, acute kidney injury, anemia, hypoproteinemia, pleural effusion, arthralgia, and lymphadenopathy following an acute *Brucella* infection. Renal biopsy revealed mesangial proliferative immunoglobulin A (IgA) nephropathy with partial crescents, classified as M1E0S0T0C2 according to the Oxford classification, in conjunction with *Brucella* spondylitis. The patient achieved complete remission after 4 months of anti-brucellosis therapy with doxycycline, levofloxacin, and rifampicin. In this paper, we present a case study of IgA nephropathy complicated by cellular crescent lesions resulting from acute *Brucella* infection, which completely resolved following anti-*Brucella* therapy. In addition, we review previously documented cases of *Brucella*-associated glomerular disease confirmed through renal biopsy, aiming to offer a reference for clinical diagnosis and treatment.

## Introduction

Brucellosis represents the most widespread zoonotic disease globally, with its endemic regions progressively expanding, thereby constituting a major public health concern worldwide (1, 2). The highest incidence of human brucellosis is observed in Asia, particularly in China and Mongolia, which bear the greatest disease burden (3).

*Brucella*, a Gram-negative bacterium characterized as facultative intracellular, is the etiological agent responsible for brucellosis. Its primary virulence attribute lies in its ability to persist and replicate within host cells over extended periods, thereby evading detection and response by the host immune system. This capability is fundamental to sustaining its chronic infection (1, 2, 4). Humans can contract the disease through various transmission routes, including contact with infected animals and their secretions, consumption of contaminated food, and occupational exposure, among others (5, 6). The disease is often challenging to accurately diagnose and is prone to misdiagnosis (7). Human brucellosis manifests with nonspecific clinical symptoms and is a systemic disease that can affect multiple organs and systems (4, 8–10), with the bones and joints being the most commonly involved (8). The urogenital system is also frequently affected, with orchitis being a common clinical presentation (9). In addition, the disease can manifest as pyelonephritis, acute interstitial nephritis, and acute kidney injury (9–12). However, glomerular diseases are relatively rare and are primarily documented in case reports within the clinical literature. Renal pathology with renal biopsy may present as immunoglobulin A nephropathy (IgAN) (13, 14), as well as diffuse proliferative, mesangiocapillary, membranoproliferative, and crescentic glomerulonephritis (15–20), each with different clinical presentations. Both acute and chronic *Brucella* infections can result in glomerular lesions. In this paper, we report the first case of IgAN with cellular crescentic lesions secondary to acute *Brucella* infection in our center.

## Case report

A 45-year-old man, who worked as a teacher with no past kidney medical history, was hospitalized on September 13, 2023, at the Department of Nephrology, Shenzhen Second People’s Hospital. On September 1, 2023, 12 days prior to admission, the patient presented with a high fever exceeding 39°C, accompanied by chills, generalized myalgia, arthralgia, hyperhidrosis, nausea, and vomiting. Approximately 20 years ago, the patient experienced an abrupt onset of left-sided deafness. Furthermore, he has a 3-year history of untreated hypertension, with systolic blood pressure readings consistently around 140 mmHg.

Upon admission, a physical examination was conducted, which revealed the following vital signs: blood pressure at 145/90 mmHg, pulse rate at 83 bpm, and body temperature at 38.9°C. Clinical examination indicated the absence of edema and no abnormal findings. Laboratory analyses demonstrated a white blood cell count of 20.5 × 10^9^/L, hemoglobin level of 130 g/L, serum albumin concentration of 31.1 g/L, and blood creatinine levels ranging from 220 to 330 μmol/L. The hypersensitivity C-reactive protein (CRP) was measured at 97.3 mg/L, and procalcitonin (PCT) was 1.06 ng/ml. Urinalysis revealed proteinuria at 2+ mg/dl and red blood cells at 2+ per high-power field (HPF), with a 24-h urinary protein excretion of 1.51 g. The erythrocyte sedimentation rate was 35 mm/h. Tests for *Plasmodium sanguinis*, dengue antigen/antibody, urine bacterial culture, and tuberculosis infection T cells were all within normal limits. In addition, tests for anti-neutrophil cytoplasmic antibody (ANCA), anti-glomerular basement membrane (GBM) antibody, anti-double-stranded DNA (dsDNA), and antinuclear antibody (ANA) were negative. Complement C3 was 0.92 g/L and C4 was 0.22 g/L. The patient was negative for hepatitis B, hepatitis C, syphilis, and HIV.A monoclonal protein fraction was not detected in the serum protein electrophoresis. The virology assays for cytomegalovirus, hepatitis viruses, Epstein–Barr virus, and herpes simplex virus returned negative results. Ultrasonographic evaluation of the kidneys indicated normal morphology and size. The cardiac ultrasound findings revealed an enlargement of the left atrium, while the morphology and structure of the cardiac valves appeared normal. Computed tomography (CT) of the lungs showed no evidence of infection or effusion. The patient was administered piperacillin/tazobactam for anti-infective therapy over the course of 1 week. However, symptoms of fever, nausea, vomiting, and arthralgia recurred. The patient presented with a body temperature of 38.3°C and reported symptoms of urinary urgency, frequency, gross hematuria, and a cough without expectoration.

Meropenem was given as anti-infective treatment. A subsequent physical examination revealed no tenderness at the ureteral points, no percussion pain in the renal area, and no edema in the extremities. Repeat laboratory analyses indicated a white blood cell count of 14.73 × 10^9^/L, hemoglobin level of 96.0 g/L, and blood creatinine concentration of 150.5 μmol/L. Urinalysis demonstrated proteinuria at 2+ mg/dl, red blood cells at 3+ per HPF, and negative urine white blood cells. The blood and bone marrow cultures showed no bacterial growth. The patient was also negative for influenza A/B virus and novel coronavirus.

An abdominal ultrasound revealed enlarged kidneys, with the left kidney measuring 130 mm × 61 mm and the right kidney measuring 132 mm × 57 mm. Enlarged lymph nodes were identified in the right axillary region and the bilateral groin area. A cardiac ultrasound showed no valvular vegetation. CT of the lungs indicated inflammation in both lungs and a small amount of effusion. Abdominal CT demonstrated exudative changes around both kidneys, likely due to inflammation. A bone marrow biopsy revealed markedly active hyperplasia in the granulocyte and megakaryocyte lineages, with decreased erythroid hyperplasia; scattered and clustered platelets were readily observed. The renal biopsy pathology indicated mesangial proliferative IgAN with some glomerular crescent formation, classified as M1E0S0T0C2 according to the Oxford classification. Immunofluorescence analysis showed IgG negativity, IgA (++), IgM (+), C3 (+), and C1q negativity, deposited in the mesangial region. Examination under a light microscope revealed 11 glomeruli, with no evidence of glomerular sclerosis or stage sclerosis.

**Figure 1:**
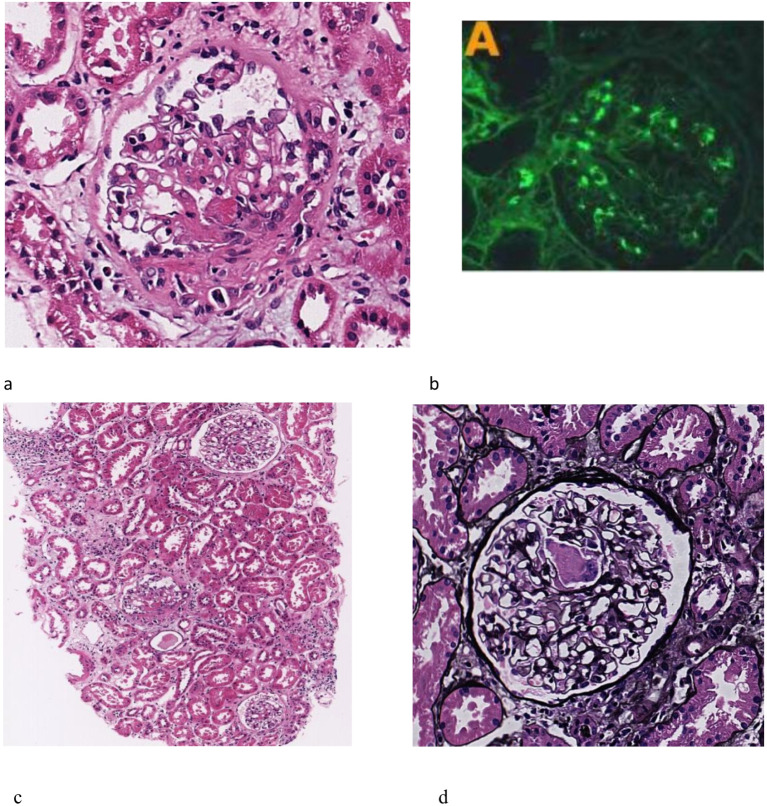
Renal biopsy findings. **(a)** mild proliferation of mesangial cells and matrix (HE stain, 100×). **(b)** fibroid necrosis of segmental capillary vessels (PAS stain, 400×). **(c)** small cell crescents were seen. (HE stain, 400×). **(d)** Immunofluorescence showed Immunoglobulin A (IgA) is deposited in the mesangial region, exhibiting a comma-like distribution pattern, with a deposition intensity of (++). The renal biopsy pathology revealed mesangial proliferative IgA nephropathy with some glomerular crescent formation, Oxford classification was M1E0S0T0C2, combined with brucellary spondylitis. 11 glomeruli were seen under light microscope, but no glomerular sclerosis and stage sclerosis were observed. The glomeruli were of varying size and size, mild proliferation of mesangial cells and matrix, no obvious endothelial cell proliferation, fuchsinophilic material deposition in the mesangial area, fibroid necrosis of segmental capillary vessels in 2 glomeruli, and 5 small cell crescents were seen. The renal interstitial focus is strong, inflammation and vacuolar degeneration, with mild fibrosis and slight thickening of the arteriole wall. Electron microscope show no obvious thickening of the glomerular basement membrane, segmental fusion of the foot processes, and a small amount of electron-dense material can be seen in a few mesangial areas.

The glomeruli exhibited variability in size, accompanied by a mild proliferation of glomerular mesangial cells and matrix and deposition of fuchsinophilic material in the mesangial area, without evident endothelial cell proliferation. There was fibroid necrosis in segmental capillary vessels observed in two glomeruli, as well as the presence of five small cellular crescents. Vacuolar degeneration of renal tubular epithelial cells was observed. The renal interstitium demonstrated pronounced inflammation, along with mild fibrosis and a slight thickening of the arteriolar walls. Electron microscopy revealed no significant thickening of the glomerular basement membrane, segmental fusion of foot processes, and a small quantity of electron-dense material in select mesangial regions. There were no obvious subepithelial electron-dense deposits (see **Figure 1**).

Upon reviewing the patient’s medical history, it was noted that the patient had been in contact with raw mutton 2 weeks prior to disease onset, had sustained damage to the skin on his hands, and had consumed cooked mutton. Further diagnostic tests indicated a positive result (+++) for the Rose Bengal test (RBT) and a positive result (1:200+++) for the standard agglutination test (SAT). Magnetic resonance imaging (MRI) revealed scattered patchy, nodular, proton density (PD) fat-suppressed hyperintense signals in the thoracic vertebrae (T4, T5, T7, T9, and T11) and the first lumbar vertebra (L1), with significant lesions observed in L1, which were considered to be infectious in nature, leading to a diagnosis of *Brucella* spondylitis. Cerebrospinal fluid (CSF) analysis showed no significant abnormalities. The results of the laboratory tests are presented in **Table 1**.

**Table 1:** Laboratory tests at beginning and at follow-up.

Laboratory tests	Disease course (days)										Laboratory tests
Blood work-up	3	4	6	11	13	16	19	50	120	350	Normal range
Albumin (g/d)			31.1			28.2	34.4	41.6			40-55
Serum creatinine (μmol/l)	220	330.3	228.6	162.1	150.1	144.2	129	95.4	87.5	86.2	57-97
eGFR (ml/min/1.73m^2^)	31.73	19.40	30.29	45.91	50.39	52.89	60.52	86.58	96.13	97.89	90-120
BUN (mmol/l)	12.7	18.7	10	5.9	5.1	4.7	4.5	5.8	6.31	6.3	3.1-8.0
Uric acid (μmol/l)	433	506.5	289.7	210	252.2	256.2	242.1	483.6	490	336.4	208-428
WBC count (1000cells/mm3)	20.58	20.68	10.32	10.25	14.73	14.6	11.28	6.27		5.77	3.5-9.5
N (%)	89.3	89.3	79.6	75.0	84.6	77.9	77.3	48.4		56.2	40-75
HB (g/L)	130	120	100	97	96	87	91	105		130	130-175
Platelet count (1000 cells/mm3)	199	168	161	273	256	228	272	179		206	125-350
CRP (mg/L)	97.32		34.89		33.48	48.14	22.31				0-5
PCT	1.06		0.14		0.06	0.07	0.04				<0.05
Urinalysis											
Urine Specific Gravity	1.013		1.006		1.01	1.003	1.004	1.012	1.023	1.012	1.003-1.030
Proteinuria	2+		2+		2+	1+	1+	N	1+	N	negative
Hematuria (RBC/HPF)	1+		3+		3+	2+	2+	69.7	2-4/hp	N	negative
24-h urinary protein (g/d)			1.52	1.01	1.49					0.03	0-0.15

eGFR, estimated glomerulofiltration rate; The CKD-EPI (the Chronic Kidney Disease Epidemiology Collaboration) equation was used for evaluation.

The patient was diagnosed with brucellosis, *Brucella* spondylitis, and brucellosis-related renal impairment. The decision was made to prioritize the treatment of brucellosis, with subsequent treatment plans to be determined based on the efficacy of the initial intervention. A therapeutic regimen consisting of rifampicin (600 mg/day), doxycycline (200 mg/day), and levofloxacin (500 mg/day) was administered, with treatment duration of 4 months. Remarkably, after 7 days of treatment, the patient’s clinical symptoms, including the fever and musculoskeletal pain, were alleviated, although significant sweating persisted. After 1 month, the patient’s sweating had also improved, and the kidney function tests returned to normal. Repeat laboratory analyses revealed a hemoglobin level of 105 g/L, blood albumin of 41.6 g/L, and creatinine at 95.4 μmol/L. Urinalysis indicated negative proteinuria, 69 red blood cells per HPF, and negative urine white blood cells. After 3 months, the creatinine levels decreased to 87.5 μmol/L, and urinalysis showed positive proteinuria, two to four red blood cells per HPF, and a specific gravity of 1.023. The *Brucella* antibody tube agglutination test was negative. At 11 months of follow-up, the patient’s blood creatinine was 86 μmol/L, and urinalysis indicated resolution of the proteinuria and red blood cells.

## Discussion

In this report, we present the case of a 45-year-old male teacher with no prior history of renal disease. The patient reported exposure to raw mutton 2 weeks prior to the onset of symptoms, during which time the skin on his hands was compromised. It is hypothesized that the patient contracted an infection through direct contact with *Brucella*-contaminated raw mutton. Subsequently, the patient developed glomerulonephritis following acute *Brucella* infection, which affected multiple organ systems. Renal biopsy revealed mesangial proliferative IgAN with some glomerular crescent formation, classified as M1E0S0T0C2 according to the Oxford classification, in conjunction with *Brucella* spondylitis. Following a 4-month regimen of anti-brucellosis therapy, which included doxycycline, levofloxacin, and rifampicin, the patient achieved complete remission. Post-treatment, both the urinalysis abnormalities and the renal function were fully restored. We hypothesize that the patient’s IgAN with cellular crescent formation was induced by the *Brucella* infection.

*Brucella* infection affects multiple organ systems, with patients often presenting with nonspecific symptoms such as fever, fatigue, night sweats, vomiting, arthralgia, myalgia, hepatomegaly, and lymphadenopathy (4). The literature indicates that both acute and chronic *Brucella* infections can lead to *Brucella* nephropathy, which is characterized by a wide range of clinical manifestations, predominantly resulting in acute kidney injury (16–20). The clinical presentations may include gross hematuria and significant proteinuria, with some patients developing nephrotic syndrome, potentially accompanied by anemia, hypocomplementemia, pleural effusion, and cardiac valve vegetations (21). In addition, two cases of chronic *Brucella* infection have been associated with cryoglobulinemia and ANCA positivity (20). In our case, the patient exhibited renal manifestations alongside anemia, pleural effusion, and, notably, *Brucella* spondylitis, without evidence of hypocomplementemia or valvular heart disease. These findings suggest that, in patients with *Brucella* nephropathy, it is crucial to consider and exclude the possibility of *Brucella* spondylitis and endocarditis.

Prior studies have demonstrated that the renal biopsy pathology results show that, in addition to IgAN, the renal pathology associated with *Brucella* nephropathy may encompass minimal lesions, diffuse, mesangial, and membranous proliferative glomerulonephritis, and crescentic glomerulonephritis, among others (see **Table 2**).

**Table 2:** Summary of glomerular disease caused by *Brucella* infection.

Author	Magazine/Year	Age(y)/sex	Clinical manifestations	Renal biopsy	Medication/Course	Outcome
Siegelmann N, (13)	Postgrad Med J. 1992	43/F	NS, Cr 229μmol/l, proteinuria (10.5g/d), Hematuria, Anemia,	IgA nephropathy	DOX and RIF, 6 W	recovery
Nunan TO, (14)	Br Med J (Clin Res Ed). 1984	20/M	Cr 167μmol/l, proteinuria (0.6-2,8g/d), Hematuria	mesangial IgA nephropathy	DOX and RIF, 3 M.	recovery
Parlak E (16),	Trop Doct. 2020	58/M	NS, hepatomegaly, Cr 9.24μmol/l, much protein, anemia	diffuse proliferative glomerulonephritis	DOX and RIF, 6 W	recovery
Altiparmak M R (17),	Scandinavian Journal of Infectious Diseases. 2002	33/M	Cr 0.9mg/dl, Hematuria, proteinuria(1.4g/d), decreased C3, congenital bicuspid aortic valve, hepatomegaly, anemia, heart failure	mesangiocapillary glomerulonephritis	DOX and RIF, 6 W	proteinuria; Cr. Cl.: 94 ml/min
Ardalan M R (22),	Nephrology dialysis transplantation, 2006	28/M	Cr 4mg/dl, proteinuria(1g/d), Hematuria, decreased C3, Ventricular septal defect	mesangiocapillary glomerulonephritis	RIF and DOX, MP 500 mg/d*3, oral PRED, treatment Course unknown	Recovery
Shebli HM (19),	Saudi J Kidney Dis Transpl. 2021	46/F	ALB 19.5g/L, Cr5.53 mg/dL, PCR 1.5g/g, Hematuria, anemia, Nephromegaly, hepatosplenomegaly	rapidly progressive crescentic glomerulonephritis	DOX and RIF, 4 W	Recovery
Sabanis N (23),	Saudi journal of kidney diseases and transplantation. 2016	53/M	NS, Cr 1.3mg/dl, proteinuria(12g/d)	minimal change disease	RIF and DOX, 6 W; PRED 12 M.	Recovery
Senturk BG (24),	Clin Case Rep Int. 2021	67/M	Cr 2.58mg/dl, Hematuria, No proteinuria, decreased C3, ischemic heart disease, tuberculosis osteomyelitis	immune complex focal segmental necrotizing glomerulonephritis	DOX and RIF, 6 W; PRED, CTX, AZA 6 M.; Cs+RTX, DOX, RIF, CRO	no complaints and Cr 1.44 mg/dl
Doregatti C (25),	Nephron. 1985	31/M	NS, hematuria	acute glomerolunephritis	TETRA, treatment Course unknown	Recovery
Elzouki AY (21),	Pediatr Nephrol. 1996	6/M	Cr 256μmol/l, proteinuria (2+), Hematuria, anemia, hepatomegaly, heart failure, vegetation on the mitral valve	glomerulonephritis and renal vasculitis	TMP–SMX, RIF, STREP, 8 W; PRED 2 mg/kg.d	Recovery
Ustun I (12),	South Med J. 2005	17/M	Cr 3.6mg/dl, Hepatomegaly, hematuria, proteinuria (1.2 g/d).	diffuse tubulointerstitial nephritis and diffuse proliferative glomerulonephritis	RIF and DOX, 4 W	Recovery
Kocyigit I (26),	Ren Fail, 2011	20/M	Native Aorta Valve Endocarditis and Myopericarditis, NS, hematuria, AKI, anemia, decreased C3	proliferative glomerulonephritis, Acute Postinfectious Glomerulonephritis	RIF, DOX, CRO, Aortic valve replacement	Recovery
Yang X, (20)	BMC Infectious Diseases. 2023	49/M	chronic brucellosis, NS, CHF, anemia, livedoid, PR3 -ANCA, cryoglobulinemia, Cr 203μmol/l, proteinuria 2.1 g/d	endocapillary proliferative Glomerulonephritis, crescent formation,	PRED 50mg/d; RIF and DOX, 6 M.	CR 109 μmol/l, Proteinuria 0.47g/d,
Provatopoulou S (18),	Kidney Research and Clinical Practice. 2018	39/M	chronic brucellosis, NS, hepatomegaly, decreased C3, Cryoglobulinemia	Membranoproliferative glomerulonephritis	DOX, RIF, and TMP–SMX; After 5 M., PRED 0.5 mg/kg.d, 3 M.	partial remission; 28 M.: eGFR 47 ml/min, NS

NS, nephrotic syndrome; Cr.Cl., creatinine clearance; eGFR, estimated glomerular filtration rate; GN, glomerulonephritis; TIN, tubulointerstitial nephritis; IgAN, IgA nephropathy; MCGN, mesangiocapillary glomerulonephritis; DOX, doxycycline; RIF, rifampicin; TETRA, tetracycline; CRO, ceftriaxone; STREP, streptomycin; TMP–SMX, trimethoprim–sulfamethoxazole/co-trimoxazole; PRED, prednisone; Cs, corticosteroids; MP, methylprednisolone; RV, renal vasculitis. NA, not available; Anemia; Hematuria; proteinuria; CHF, congestive heart failure, RTX, rituximab; AZA, azathioprine; W, Week; M., months;

In our case, the condition manifested following an acute *Brucella* infection. The renal biopsy pathology revealed mesangial proliferative IgAN, which was characterized by two instances of glomerular segmental capillary fibrinoid necrosis, five small cell crescents, and a focal area of strong interstitial inflammation. Previous literature has documented two instances of IgAN attributed to acute *Brucella* infection (13, 14). Compared with these earlier cases, our patient exhibited more pronounced renal pathological changes. Notably, repeated renal biopsies in patients with *Brucella*-induced IgAN, even after treatment-induced remission, have demonstrated persistent proliferation in the glomerular mesangial region and continued IgA deposition (14).

In our case, the renal pathology also indicated crescentic lesions. Previous studies have reported that acute *Brucella* infection can lead to rapidly progressive crescentic glomerulonephritis (19), accompanied by acute inflammatory cell infiltration. Previous studies have documented that chronic brucellosis infection is associated with the development of cryoglobulinemia and ANCA-associated vasculitis (20). The renal biopsy of the patient revealed intracapillary proliferative glomerulonephritis with a limited presence of crescents. This finding indicates that *Brucella* infection may induce alterations in glomerular crescent formation.

Currently, there are no established guidelines or recommendations for the treatment of *Brucella* nephropathy, with the existing literature limited to case reports. In our case study, the patient received a combination therapy of doxycycline, levofloxacin, and rifampicin over a 4-month period due to complications from *Brucella* spondylitis and IgAN. The clinical symptoms of the patient showed improvement within 1 week of initiating treatment. After 1 month, the renal function normalized, the proteinuria resolved, and microscopic hematuria decreased. By the end of the 4-month treatment, the hematuria had also resolved. In two previously documented cases of IgAN following *Brucella* infection, the renal impairment fully resolved after anti-*Brucella* therapy, with treatment durations of 6 weeks and 3 months, respectively (13, 14). Our observations indicate that while the renal function and proteinuria improved rapidly following anti-*Brucella* treatment, the hematuria resolved more gradually. This slower resolution of the hematuria may be attributed to the persistent deposition of IgA immune complexes in the mesangial region.

The treatment outcomes for renal glomerular disease secondary to acute brucellosis indicate that the majority of patients achieve complete remission following anti-brucellosis therapy, typically over a period of 6 weeks to 3 months (12, 16, 25). Nonetheless, some patients continue to exhibit persistent proteinuria despite antibacterial treatment alone (17). A subset of these patients has been successfully treated with a combination of corticosteroids and anti-brucellosis agents, leading to full resolution of the glomerular lesions (22, 23). In cases where patients present with concurrent aortic valve endocarditis and pericarditis due to *Brucella* infection, treatment regimens involving rifampicin, doxycycline, and ceftriaxone (26), or a combination of anti-brucellosis therapy and hormonal therapy (21), have resulted in complete clinical remission. Furthermore, for *Brucella*-associated rapidly progressive glomerulonephritis, a therapeutic strategy incorporating anti-*Brucella* agents and immunosuppressive therapies, such as corticosteroids, cyclophosphamide, and azathioprine, has been employed. In certain cases, additional interventions, including rituximab and plasma exchange, have also been utilized (24, 27). These strategies yielded favorable outcomes, with the patients demonstrating good prognosis.

Currently, there are two documented cases of glomerular disease attributed to chronic *Brucella* infection (18, 20). The clinical manifestations in these patients included nephrotic syndrome, significant renal function impairment, and the presence of cryoglobulinemia. In one case, the renal pathology revealed membranoproliferative glomerulonephritis (18). The patient was treated with doxycycline, rifampicin, and trimethoprim/sulfamethoxazole. After 5 months, the patient’s urinary protein level was 14 g/day, prompting the administration of a reduced dose of corticosteroids, which resulted in partial remission of the proteinuria. The second patient exhibited coexisting cryoglobulinemia and ANCA-associated vasculitis (20) and received treatment with prednisone, doxycycline, and rifampicin for 6 months, leading to the rapid alleviation of all symptoms.

The current therapeutic strategies for *Brucella* nephropathy primarily target various renal pathological types and associated complications. Different treatment modalities are employed, and most yield favorable prognoses. Nevertheless, there is presently no standardized treatment protocol for *Brucella* nephropathy (28–30). The glomerular disease induced by *Brucella* infection is believed to be associated with the production of circulating immune complexes and antibodies (31). The underlying mechanism remains unclear, necessitating further clinical investigation to elucidate both the pathogenesis and the optimal treatment approaches.

## Data Availability

The original contributions presented in the study are included in the article/supplementary material. Further inquiries can be directed to the corresponding author.
